# Pediatric DOCK8 Deficiency Beyond Infections and Atopy: An Immunoactinopathy with Immune Dysregulation and Multisystem Involvement

**DOI:** 10.3390/children13091226

**Published:** 2026-09-10

**Authors:** Figen Çelebi Çelik, Necmi Can Yüksel, Ömer Akçal, Emre Fırat, Aymen Hişmioğulları, Soner Günder, Gülçin Kaymakoğlu, Nesrin Gülez, Ferah Genel

**Affiliations:** Division of Allergy and Immunology, Department of Pediatrics, Dr. Behcet Uz Child Disease and Surgery Training and Research Hospital, Izmir Faculty of Medicine, University of Health Sciences, İzmir 35210, Türkiye; necmican.yuksel@saglik.gov.tr (N.C.Y.); omer.akcal@saglik.gov.tr (Ö.A.); emre.firat2@saglik.gov.tr (E.F.); a.hismiogullari@saglik.gov.tr (A.H.); soner.gunder@saglik.gov.tr (S.G.); gulcin.ciftci2@saglik.gov.tr (G.K.); nesrin.gulez@sbu.edu.tr (N.G.); ferah.genel@sbu.edu.tr (F.G.)

**Keywords:** DOCK8 deficiency, immunoactinopathy, actin cytoskeleton, inborn errors of immunity, immune dysregulation, hyper-IgE syndrome, hematopoietic stem cell transplantation, pediatric immunodeficiency

## Abstract

**Highlights:**

**What are the main findings?**

- This tertiary-center cohort supports viewing pediatric DOCK8 deficiency as an actin cytoskeleton-related IEI with overlapping infectious, allergic, immune-dysregulatory, and systemic manifestations.
- Beyond eczema and recurrent infections, the cohort included cytopenias, autoimmunity, organomegaly, hepatobiliary disease, malignancy, and selected vascular/systemic complications.

**What are the implications of the main findings?**

- Recognizing DOCK8 deficiency as an actin cytoskeleton-related IEI with immune dysregulation may help clinicians interpret severe atopy, viral skin disease, autoimmunity, and systemic findings within a single disease framework.
- Timely genetic diagnosis and early hematopoietic stem cell transplantation evaluation are critical to prevent cumulative organ damage and improve long-term outcomes.

**Abstract:**

**Background/Objectives**: Dedicator of cytokinesis 8 (DOCK8) deficiency is an autosomal recessive combined immunodeficiency and an actin cytoskeleton-related inborn error of immunity characterized by severe atopy, recurrent infections, and broad immune dysregulation. We aimed to describe the clinical, immunological, genetic, treatment-related, and outcome features of children with genetically confirmed DOCK8 deficiency, emphasizing immune dysregulation and systemic involvement. **Methods**: We retrospectively reviewed 17 pediatric patients from 14 unrelated kindreds with genetically confirmed DOCK8 deficiency followed at a tertiary pediatric immunology center between 2005 and 2026. Demographic data, infectious and allergic manifestations, autoimmune and hematological findings, organ involvement, malignancy, laboratory parameters, genetic findings, hematopoietic stem cell transplantation (HSCT) status, and survival outcomes were analyzed descriptively. **Results**: Twelve patients were male (70.6%), and parental consanguinity was present in 13 patients (76.5%). The median age at symptom onset was 5 months (IQR, 3–10), whereas the median age at diagnosis was 44 months (IQR, 23–56). A history of eczema was documented in all patients. Recurrent skin infections occurred in 16 patients (94.1%), recurrent pneumonia in 13 (76.5%), mucocutaneous candidiasis in 10 (58.8%), and cytomegalovirus infection in four (23.5%). Confirmed autoimmune manifestations were documented in two patients, including autoimmune hepatitis and autoimmune hemolytic anemia. Malignancy occurred in two patients: gastrointestinal stromal tumor and cutaneous squamous cell carcinoma. Additional uncommon systemic manifestations included sclerosing cholangitis, giant aortic aneurysm, and chronic pancreatitis. HSCT was performed in 12 patients (70.6%); complete clinical recovery was achieved in 11, whereas one patient died after transplantation. Four of five non-transplanted patients died during follow-up. **Conclusions**: Pediatric DOCK8 deficiency showed an early-onset, severe, multisystem phenotype. Beyond infections and atopy, immune dysregulation-related and systemic manifestations were clinically important. Favorable HSCT outcomes were consistent with previous evidence supporting early molecular diagnosis and timely transplant evaluation.

## 1. Introduction

Inborn errors of immunity (IEIs) are now understood as disorders with overlapping clinical dimensions rather than isolated defects of host defense. Infection susceptibility may coexist with allergy, autoimmunity, lymphoproliferation, malignancy, inflammation, and syndromic or systemic features within the same monogenic condition [1]. This multidimensional view is particularly relevant for immune-related actinopathies, in which impaired actin cytoskeleton regulation affects immune-cell migration, survival, synapse formation, tissue surveillance, and immune tolerance [2,3].

Dedicator of cytokinesis 8 (DOCK8) deficiency is an autosomal recessive combined immunodeficiency and a prototypic actin cytoskeleton-related IEI. DOCK8 dysfunction disrupts cytoskeletal remodeling and immune synapse integrity, thereby affecting T cells, B cells, natural killer cells, dendritic cells, and regulatory T-cell function [3,4,5,6,7,8]. Clinically, the disease often begins in infancy with severe eczema, recurrent respiratory and cutaneous infections, eosinophilia, elevated IgE, and allergic disease. However, longitudinal follow-up has shown that the phenotype may extend to cytopenias, autoimmunity, hepatobiliary involvement, vasculopathy, lymphoproliferative or organomegaly-related findings, and malignancy [9,10,11,12,13,14,15].

This broader phenotype has therapeutic implications. Hematopoietic stem cell transplantation (HSCT) is the only established curative treatment and may improve infections, eczema, allergic manifestations, immune function, and survival, particularly when performed before irreversible organ damage or malignancy develops [16,17,18,19]. Therefore, timely recognition of DOCK8 deficiency requires attention not only to eczema and recurrent infections, but also to accompanying immune dysregulation and systemic disease burden.

In this study, we describe 17 pediatric patients from 14 unrelated kindreds with genetically confirmed DOCK8 deficiency. We aimed to characterize their clinical, immunological, genetic, treatment-related, and outcome features, with particular emphasis on immune dysregulation as a unifying component of atopic, hematological, autoimmune, organomegaly-related, hepatobiliary, malignant, and systemic manifestations.

## 2. Materials and Methods

### 2.1. Study Design and Patients

This retrospective, single-center cohort study included children with genetically confirmed DOCK8 deficiency who were followed in the Pediatric Allergy and Immunology Department of Dr. Behçet Uz Children’s Hospital, İzmir, Türkiye, between 2005 and 2026. Patients were eligible for inclusion if they had a molecular diagnosis of DOCK8 deficiency and available clinical, immunological, genetic, and follow-up data in the hospital records. Patients with incomplete diagnostic confirmation or insufficient clinical documentation were excluded. The study was designed to describe the clinical and immunological spectrum of DOCK8 deficiency, with particular emphasis on immune dysregulation-related manifestations in addition to infectious and atopic features. The study protocol was approved by the Local Ethics Committee of Dr. Behçet Uz Children’s Hospital (Approval number: GOA-382). The study was conducted in accordance with the principles of the Declaration of Helsinki.

### 2.2. Data Collection

Demographic, clinical, laboratory, genetic, treatment-related, and outcome data were retrospectively collected from electronic and written medical records. Demographic variables included sex, age at first symptom, age at diagnosis, age at last follow-up, and parental consanguinity. Clinical variables included infectious manifestations, atopic findings, hematological manifestations, growth retardation, hepatosplenomegaly, dysmorphic facial features, dental problems, and documented infection history. Thoracic computed tomography, abdominal ultrasonography, and echocardiography findings were also recorded.

Laboratory data included complete blood count parameters, absolute lymphocyte, neutrophil, and eosinophil counts (AEC at initial evaluation and peak AEC during follow-up were recorded separately), platelet indices, serum IgG, IgA, IgM, total IgE, complement C3 and C4 levels, and lymphocyte subset analysis including CD3, CD4, CD8, CD19, and natural killer cells. The CD4/CD8 ratio was recorded when available.

Genetic data included the affected gene, molecular finding, variant category, zygosity when documented, and transcript information when available. Variant descriptions were transcribed from the available molecular genetic records. Molecular findings that were not originally reported in Human Genome Variation Society (HGVS) format were presented descriptively as recorded, without retrospective inference of unavailable nucleotide or protein-level annotations. Treatment-related data included intravenous immunoglobulin replacement, antibacterial and/or antifungal prophylaxis, antiviral treatments, hematopoietic stem cell transplantation, age at transplantation, and post-transplant outcome. Survival status at last follow-up was recorded as the principal outcome variable.

### 2.3. Definition of Immune Dysregulation-Related Phenotype

For the purpose of this study, clinical findings were evaluated within overlapping phenotypic domains rather than mutually exclusive groups. Infectious manifestations included recurrent pneumonia, recurrent skin infections or abscesses, mucocutaneous candidiasis, cytomegalovirus infection, herpesvirus-related disease, HPV-related disease, and other documented bacterial, fungal, viral, or mycobacterial infections.

Immune dysregulation-related manifestations included allergic/atopic disease, autoimmune manifestations, cytopenias, lymphoproliferative or organomegaly-related findings, and malignancy. Allergic/atopic manifestations included eczema, asthma, anaphylaxis, food allergy and allergic sensitization. Autoimmune manifestations were reported only when specifically documented in the medical records. Cytopenias were recorded separately unless an autoimmune mechanism was explicitly documented. Lymphoproliferative or organomegaly-related findings included hepatomegaly, splenomegaly, or hepatosplenomegaly.

Eosinophilia and elevated total IgE were analyzed as immunological features supporting the allergic and immune-dysregulatory phenotype. Other systemic manifestations, including growth retardation, hepatobiliary involvement, chronic pancreatitis, dysmorphic facial features, vascular and dental problems, were recorded separately as part of the multisystem disease burden.

### 2.4. Clinical Severity, Treatment and Outcome Assessment

The National Institutes of Health Hyper-IgE Syndrome (NIH-HIES) clinical score, which had been recorded during the initial diagnostic evaluation when HIES was clinically suspected, was retrospectively retrieved from the medical records and reported descriptively. Eczema severity at the time of clinical assessment was evaluated using the SCORAD (Scoring Atopic Dermatitis) index. Thoracic computed tomography findings were reviewed for chronic lung disease, including bronchiectasis, ground-glass opacities, nodular lesions, or other structural abnormalities.

The main outcomes were survival status at last follow-up and hematopoietic stem cell transplantation outcome. Among transplanted patients, outcomes were categorized as complete clinical recovery or death; non-transplanted patients were evaluated separately. Donor type and conditioning-regimen details were reported when available; missing information was not inferred. Complete clinical recovery was defined as survival with documented donor engraftment, no ongoing requirement for immunoglobulin replacement therapy, and absence of severe or recurrent DOCK8-related infections during follow-up.

### 2.5. Statistical Analysis

Statistical analyses were performed using IBM SPSS Statistics for Windows, version 22.0 (IBM Corp., Armonk, NY, USA). Given the rarity of DOCK8 deficiency and the limited cohort size, the study was primarily designed as a descriptive analysis. Categorical variables were presented as numbers and percentages. Continuous variables were summarized as median, interquartile range, and minimum–maximum values. Percentages were calculated according to the number of patients with available data for each variable, and missing data were not imputed. Clinical findings were evaluated within overlapping phenotypic domains, including infectious, allergic/atopic, autoimmune, hematological, organomegaly-related, malignant, systemic, and treatment-related manifestations. HSCT outcomes and survival status were primarily reported descriptively. In an exploratory, unadjusted analysis, mortality according to HSCT status was compared using Fisher’s exact test, and the relative risk (RR) with 95% confidence interval (CI) was calculated. A two-sided *p* value of <0.05 was considered statistically significant. Exploratory correlations among NIH-HIES score, SCORAD score, peak absolute eosinophil count, and total IgE were assessed using Spearman’s rank correlation test. Comparisons of these variables according to survival status were performed using the Mann–Whitney U test.

## 3. Results

### 3.1. Demographic and Diagnostic Characteristics

Seventeen patients from 14 unrelated families with genetically confirmed DOCK8 deficiency were included in the study. Twelve patients were male (70.6%) and five were female (29.4%). Parental consanguinity was present in 13 patients (76.5%). A family history of IEI was recorded in seven patients (41.2%); in six patients (35.3%), this involved an affected sibling or a sibling with a history suggestive of a similar disorder.

The median age at first symptoms was 5 months (IQR, 3–10; range, 1–12 months), whereas the median age at diagnosis was 44 months (IQR, 23–56; range, 6–180 months). The median diagnostic delay was 35 months (IQR, 18–46; range, 2–179 months). The median age at last evaluation was 150 months (IQR, 108–207; range, 41–248 months). The median follow-up duration was 24 months (IQR, 18–60; range, 12–204 months).

### 3.2. Clinical Phenotype and Infectious Complications

**Figure 1:**
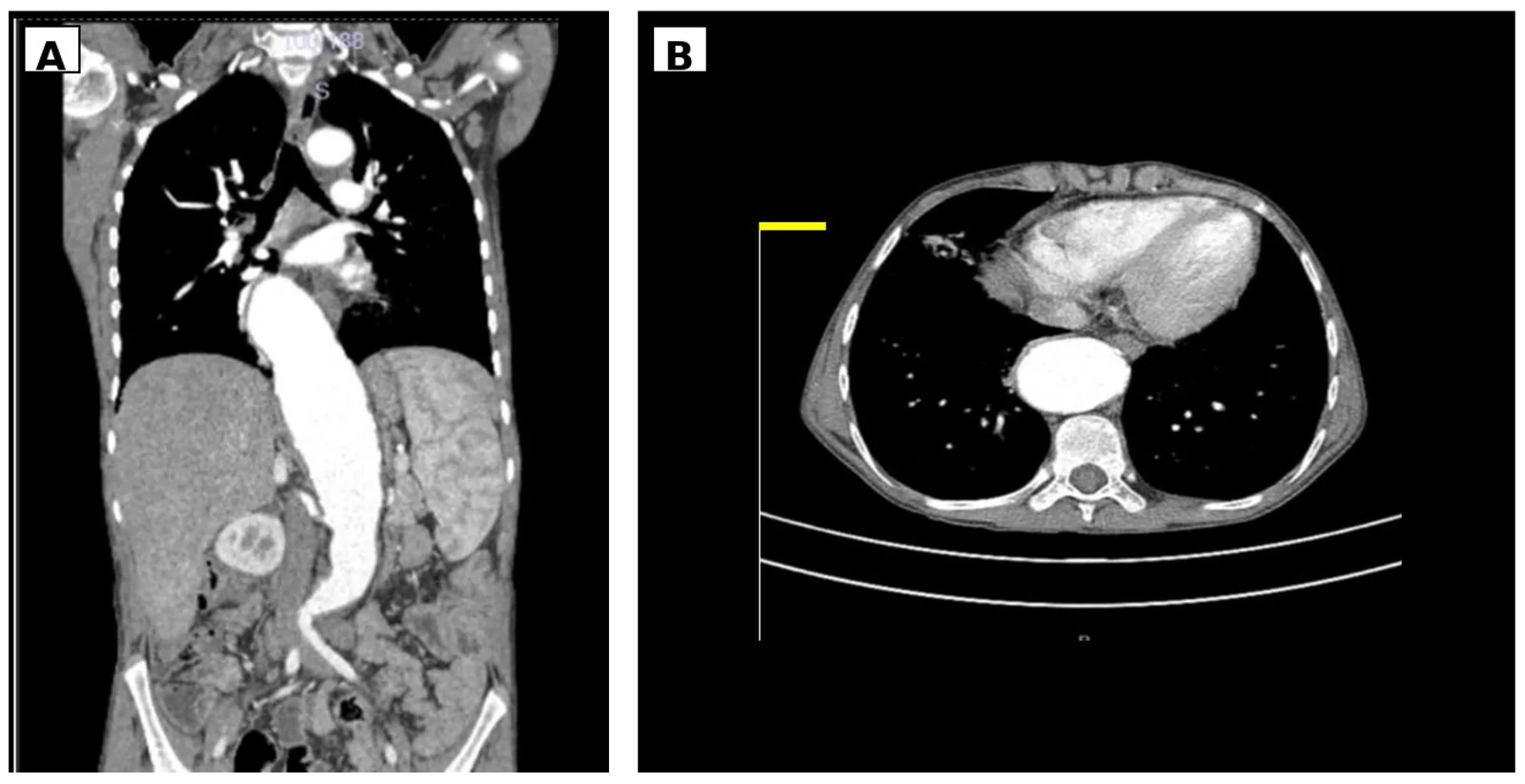
Giant aortic aneurysm in a patient with DOCK8 deficiency. (**A**,**B**) Coronal and axial thoracic CT angiography images showing a giant aortic aneurysm in one patient (F1.P1).

The cohort was characterized by early disease onset, skin findings, recurrent infections, and multisystem involvement. The median NIH-HIES score at initial diagnostic evaluation was 33 (IQR, 23–38; range, 18–52). Growth retardation was present in seven patients (41.2%), dysmorphic facial features were recorded in eight patients (47.1%), and nonspecific dental problems were documented in three patients (17.6%). Additional systemic findings observed in individual patients included chronic pancreatitis and hypertension in one patient (F11.P1), and a giant aortic aneurysm documented by thoracic CT angiography in another patient (F1.P1) (Figure 1A,B).

**Figure 2:**
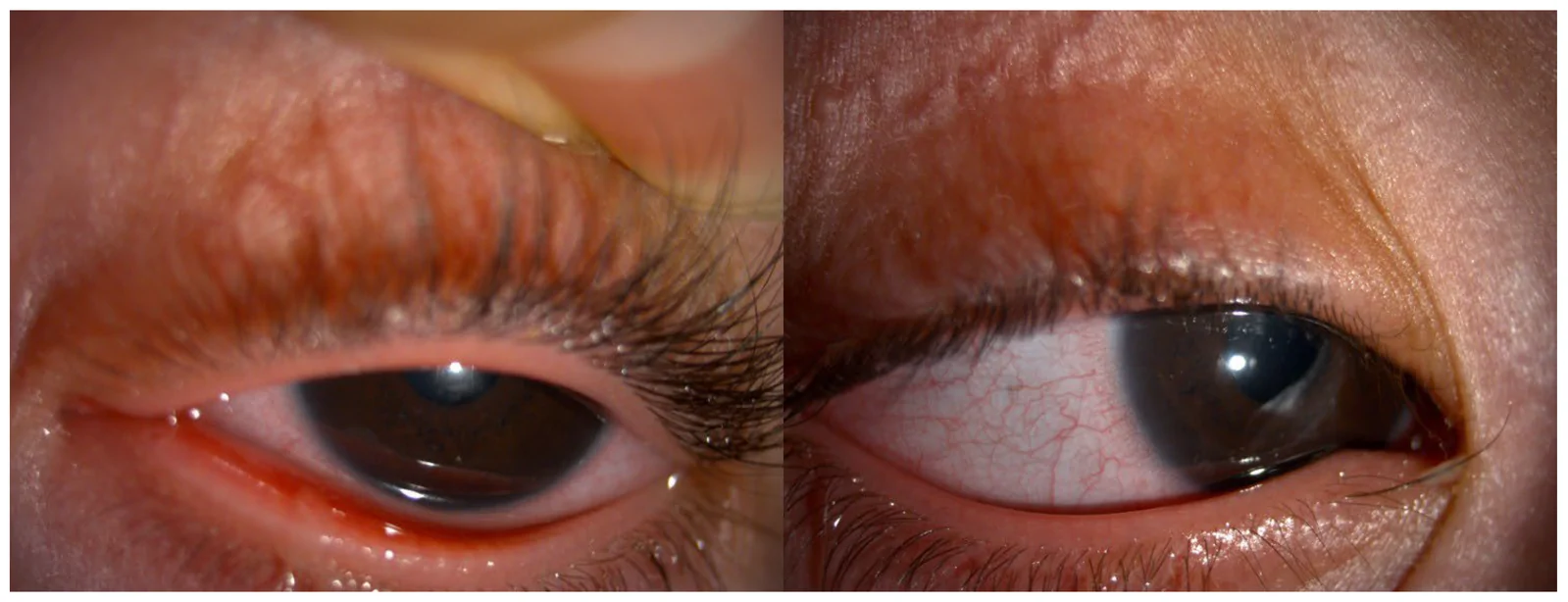
Herpetic keratitis in a patient with DOCK8 deficiency.

Infectious manifestations were frequent and clinically heterogeneous. Recurrent pneumonia was documented in 13 patients (76.5%), and recurrent skin infections were recorded in 16 patients (94.1%). Mucocutaneous candidiasis was present in 10 patients (58.8%). Cytomegalovirus infection was documented in four patients (23.5%). Herpesvirus-related manifestations included herpetic keratitis in two patients (F10.P1 and F11.P1; Figure 2), widespread herpes labialis in one of them (F11.P1), and chronic herpesvirus-related skin disease in one patient (F13.P2). HPV-related disease was present in two patients (11.8%): HPV infection in the patient with cutaneous squamous cell carcinoma (F6.P1) and HPV-related perianal and perineal condyloma in another patient (F9.P1). Pulmonary tuberculosis was recorded in one patient (F8.P2).

Representative ocular photographs showing unilateral herpetic keratitis with corneal involvement and associated periocular inflammatory changes (Figure 2).

**Figure 3:**
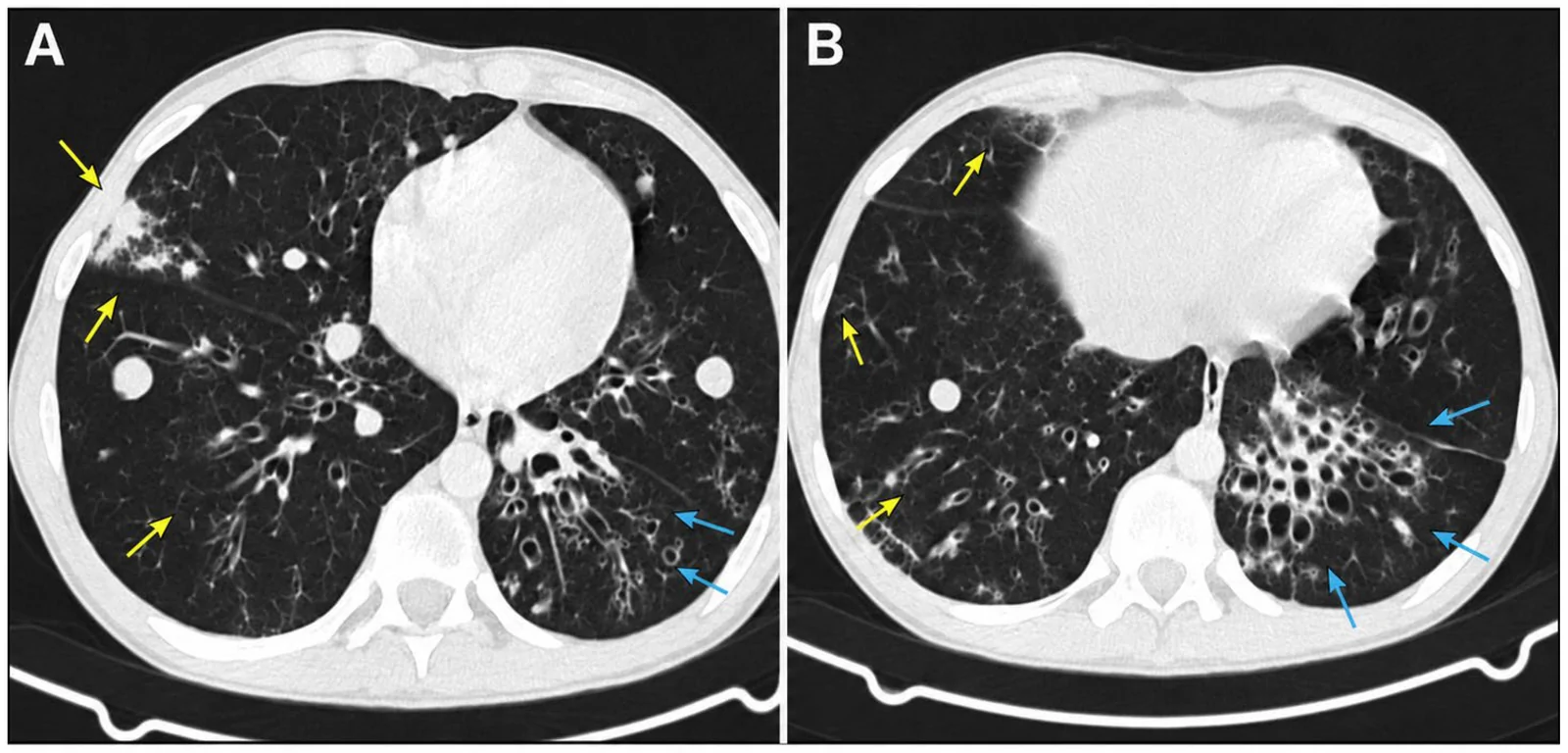
Thoracic CT findings in a patient with DOCK8 deficiency and pulmonary tuberculosis (F8.P2). (**A**,**B**) Axial thoracic CT images showing multifocal nodular and patchy parenchymal opacities in both lungs (yellow arrows) and extensive cystic/varicose bronchiectatic changes, predominantly involving the left lower lobe (blue arrows).

Thoracic computed tomography was available in 15 patients, of whom 10 (66.7%) had abnormal findings. Bronchiectasis was the most common structural lung abnormality, observed in six patients (40.0% of those with available thoracic CT data). Other thoracic findings included ground-glass opacities in two patients (13.3%), bronchiolitis obliterans in one patient (6.7%), and hypersensitivity pneumonitis in one patient (6.7%). One patient with pulmonary tuberculosis (F8.P2) had diffuse nodular pulmonary involvement accompanied by diffuse bronchiectasis on thoracic CT (Figure 3).

### 3.3. Immune Dysregulation-Related Manifestations

**Figure 4:**
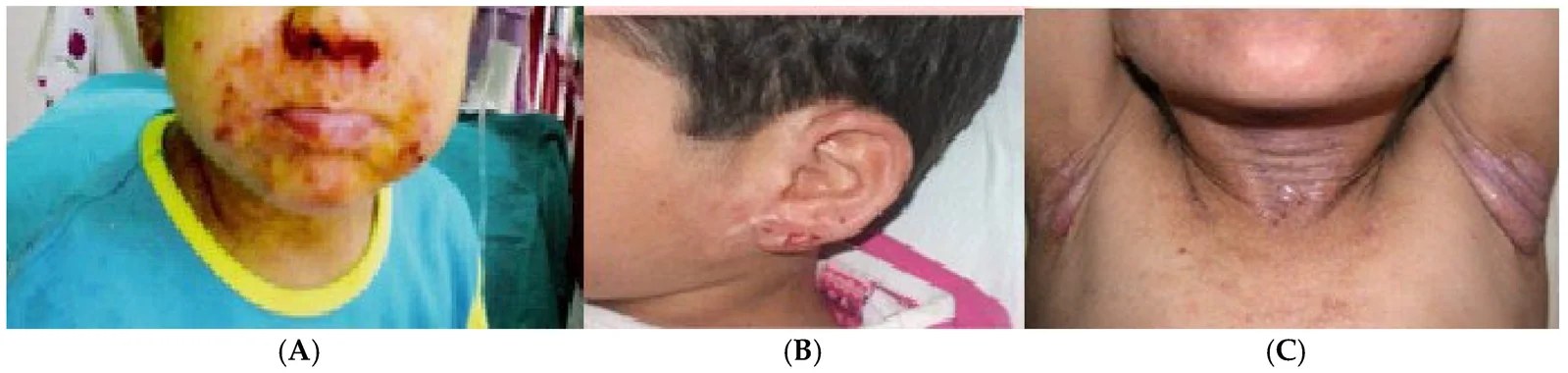
Representative eczematous manifestations in patients with DOCK8 deficiency. (**A**,**B**) Superinfected/impetiginized eczematous lesions with facial/perioral and auricular involvement. (**C**) Chronic lichenified eczema involving the neck and axillary/intertriginous regions.

Immune dysregulation-related manifestations were a prominent component of the clinical spectrum. Atopic disease was universal in the cohort. All patients had a documented history of eczema. The median SCORAD score at clinical assessment was 54 (IQR, 52–58; range, 0–78). Representative chronic lichenified and superinfected eczematous lesions are shown in Figure 4. Asthma was recorded in eight patients (47.1%), and anaphylaxis was documented in two patients (11.8%). Multiple allergic diseases were present in 10 patients (58.8%). Among patients with available sensitization data, specific IgE positivity was detected in seven of 10 patients, and food allergy was present in six of 10 patients.

Confirmed autoimmune manifestations were documented in two patients (11.8%): autoimmune hepatitis in one patient (F12.P1) and autoimmune hemolytic anemia in one patient (F1.P1). Hematological abnormalities included thrombocytopenia in one patient (F1.P1) and neutropenia in another patient (F10.P1). Cytopenias were reported separately unless their autoimmune nature was explicitly documented.

Lymphoproliferative or organomegaly-related findings were also observed. Hepatomegaly or splenomegaly was recorded clinically or radiologically in five patients (29.4%). Abdominal ultrasonography revealed abnormal findings in five patients (29.4%), including hepatomegaly in three patients and hepatosplenomegaly in two patients. One of these patients had chronic liver disease with sclerosing cholangitis (F12.P1).

Malignancy developed in two patients (11.8%): gastrointestinal stromal tumor (GIST) in one patient (F8.P2) and cutaneous squamous cell carcinoma in another patient (F6.P1). These findings demonstrate the coexistence of atopic, autoimmune, hematological, organomegaly-related, and malignant manifestations within the cohort (Table 1).

### 3.4. Laboratory and Immunological Characteristics

Marked eosinophilia and elevated IgE were prominent laboratory findings. At initial evaluation, the median AEC was 2950/µL (IQR, 1650–4030; range, 500–35,100/µL). Separately, the median peak AEC recorded during follow-up was 6460/µL (IQR, 4200–14,160; range, 1190–35,100/µL). Peak AEC exceeded 1500/µL in 16 patients (94.1%) and 5000/µL in 10 patients (58.8%) (Table 2).

The median total IgE level was 1533 IU/mL (IQR, 947–2000; range, 116–3371 IU/mL). Total IgE was above 1000 IU/mL in 12 patients (70.6%), including eight patients (47.1%) with levels above 2000 IU/mL or reported as >2000 IU/mL. The median serum IgG, IgA, and IgM levels were 1310 mg/dL (IQR, 720–1510), 118 mg/dL (IQR, 56.8–186), and 30.7 mg/dL (IQR, 26.2–42.7), respectively.

The median absolute lymphocyte count was 2783/µL (IQR, 2000–4400; range, 1180–6640/µL). Lymphocyte subset data showed a median absolute CD3+ T-cell count of 1354/µL (IQR, 969–1671), CD4+ T-cell count of 491.5/µL (IQR, 310–706.5), CD8+ T-cell count of 689/µL (IQR, 509.8–1160), CD19+ B-cell count of 678.5/µL (IQR, 368.5–1355), and natural killer cell count of 341.5/µL (IQR, 255.8–624.8). The median CD4/CD8 ratio was 0.7 (IQR, 0.6–1.2), and a CD4/CD8 ratio below 1 was observed in 10 of 16 patients with available data.

### 3.5. Genetic Findings

All 17 patients had a documented molecular diagnosis of DOCK8 deficiency. Patient-level molecular findings sufficient to define the variant category were available in 15 patients (88.2%). In two affected siblings from the same kindred (F8.P1 and F8.P2), the molecular diagnosis of DOCK8 deficiency was documented in the medical records, but the original detailed variant annotation was unavailable in the retrospective archive.

Among the 15 patients with available variant-category information, large exon-level deletions were the most frequent molecular finding, occurring in 10 patients (66.7%). Deletions involving exons 1–26 were identified in four patients from two kindreds, deletions involving exons 1–27 in three patients, and deletions involving exons 2–26, exons 2–7, and exons 1–22 in one patient each. Nonsense/stop-gain variants were documented in two patients (13.3%), missense variants in two (13.3%), and a splice-site variant in one (6.7%). Two patients had a C>G substitution reported at position 2715 of *DOCK8* cDNA near the end of exon 22, resulting in p.Ser868Ter. One patient had an A>T substitution in exon 46 at position 6068 of *DOCK8* isoform 1 cDNA (NM_203447.3), resulting in p.Asp1986Tyr. Patient-level molecular findings are summarized in Table 3.

### 3.6. Treatment and Outcomes

**Figure 5:**
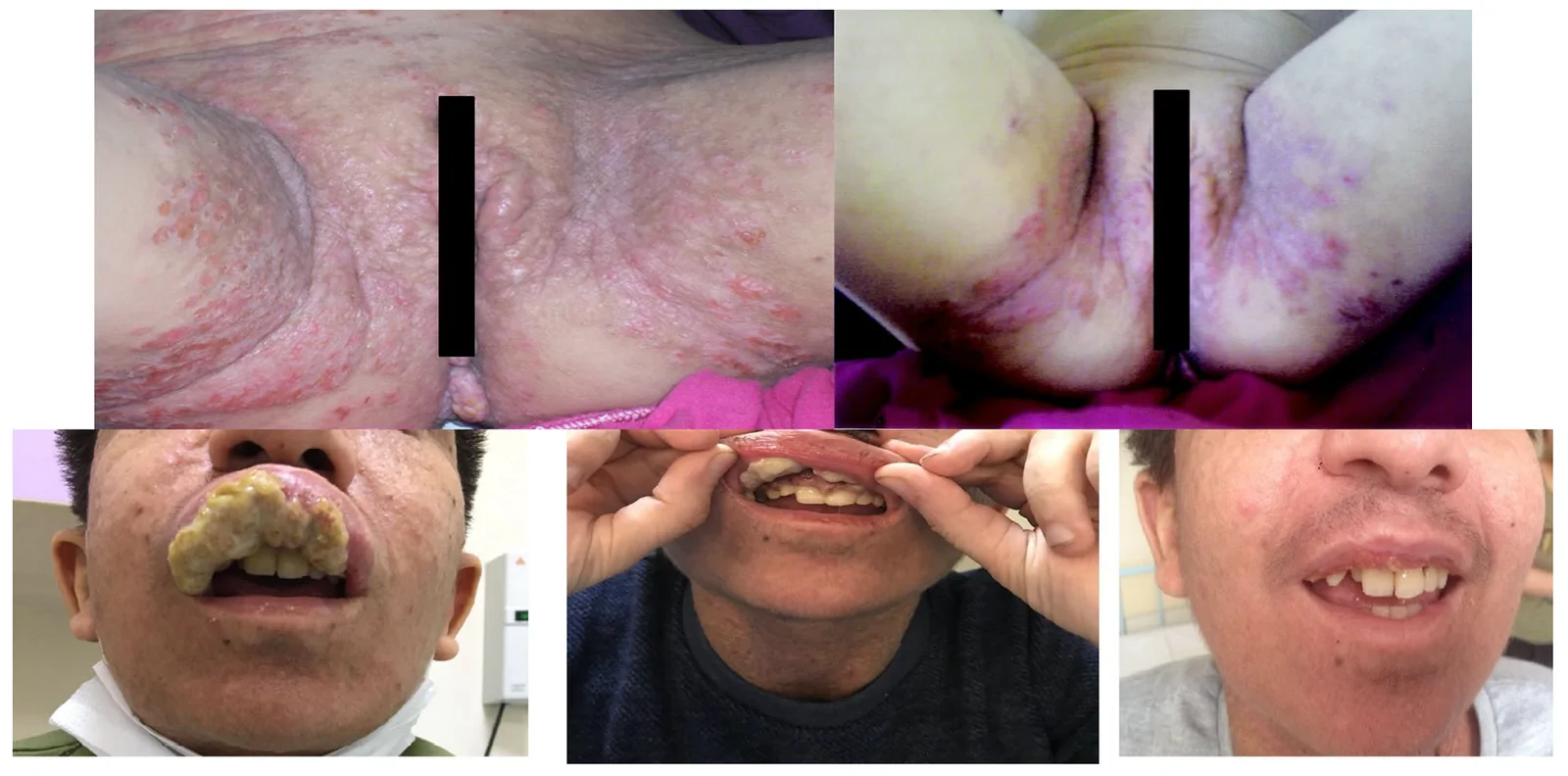
Viral mucocutaneous and treatment response in patients with DOCK8 deficiency. HPV-related perianal/perineal condylomatous lesions before and after IFN-α2b treatment in one patient (F9.P1). Widespread oral/perioral herpesvirus-related lesions and post-treatment regression following IFN-α2b, corticosteroid, and antiviral therapy in another patient (F11.P1).

All patients received intravenous immunoglobulin replacement during follow-up. In addition to intravenous immunoglobulin (IVIG) replacement and antibacterial/antifungal prophylaxis, acyclovir prophylaxis or treatment was administered to patients with herpesvirus-related disease, including herpetic keratitis, widespread herpes labialis, and chronic HSV skin infection. IFN α-2b was administered to two patients: one with widespread herpes labialis (F11.P1) and one with HPV-related condyloma (F9.P1). Clinical improvement after IFN α-2b treatment is illustrated in Figure 5. One patient with pulmonary tuberculosis received antituberculosis treatment (F8.P2).

Hematopoietic stem cell transplantation was performed in 12 patients (70.6%). The median age at transplantation was 58.5 months (IQR, 29.75–78.75; range, 18–150 months). Six patients received grafts from matched unrelated donors and one from a matched related donor; donor type was unavailable for the remaining five transplanted patients. The bone marrow or peripheral blood stem cells were used as graft sources. Conditioning was administered according to the transplantation protocols in use at the time of HSCT centers, using reduced-toxicity regimens generally designed to provide adequate marrow preparation and immunosuppression to facilitate donor engraftment. Complete clinical recovery, defined as survival with documented full donor chimerism, no ongoing requirement for immunoglobulin replacement therapy, and absence of severe or recurrent *DOCK8*-related infections, was achieved in 11 transplanted patients (91.7%) (Table 4). Among the five patients who did not undergo HSCT, three lacked a suitable donor, whereas the families of two patients declined transplantation.

At the last evaluation, 12 patients were alive and five had died, corresponding to an overall survival of 70.6%. Mortality was markedly higher among non-transplanted patients than among transplanted patients: four of five non-transplanted patients died, whereas 11 of 12 transplanted patients were alive at the last follow-up. In an exploratory analysis, the mortality rate was 80.0% in non-transplanted patients compared with 8.3% in transplanted patients (Fisher’s exact test, *p* = 0.0099). The relative risk (RR) of death in non-transplanted patients was 9.60 (95% CI, 1.40–65.94), with an absolute risk difference of 71.7 percentage points. Of the five patients who died, three non-transplanted patients died from sepsis, one non-transplanted patient died from acute respiratory distress syndrome, and the only death in the transplanted group was due to acute graft-versus-host disease during the first week after HSCT (Table 5).

Additionally, NIH-HIES score, SCORAD index, peak absolute eosinophil count, and total IgE were not significantly associated with survival status, and no significant correlations were observed among these variables.

## 4. Discussion

In this single-center pediatric cohort, DOCK8 deficiency presented as an early-onset, multisystem IEI with overlapping infectious, allergic, immune dysregulatory, and systemic manifestations. Although severe eczema and infections were prominent, the clinical spectrum also included allergy, autoimmunity, malignancy, and systemic complications. This broader clinical spectrum highlights DOCK8 deficiency not only as a combined immunodeficiency with hyper-IgE features, but also as an actin cytoskeleton-related IEI characterized by broad immune dysregulation.

In this context, DOCK8 deficiency can be viewed within the expanding spectrum of immune-related actinopathies, in which impaired actin cytoskeleton regulation disrupts immune-cell migration, immune synapse formation, cytotoxic function, tissue immune surveillance, and immune tolerance [2,3,6,7,8]. These cellular defects provide a mechanistic framework for systemic involvement observed in our cohort. This perspective is consistent with the broader concept of immunoactinopathies, which are increasingly recognized as disorders combining immunodeficiency, immune dysregulation, atopy, malignancy, hematological abnormalities, and systemic features [20,21,22,23].

Disease onset was early, whereas molecular diagnosis was substantially delayed. The marked interval between symptom onset and molecular diagnosis is clinically important, as delayed recognition may permit progressive infectious and inflammatory complications to cause irreversible organ damage. Similar diagnostic delays have been reported in other regional *DOCK8* cohorts [12,13]. The high consanguinity rate in our cohort is compatible with the autosomal recessive inheritance and with observations from populations in which consanguineous marriage is common [11,12,13]. Taken together, these findings underscore the value of early molecular testing in children with severe eczema, eosinophilia, recurrent infections, or a suggestive family history.

The allergic phenotype was one of the most consistent expressions of immune dysregulation in our patients. All patients had a documented history of eczema, with severe skin involvement observed in most cases. The frequent coexistence of eczema, allergic sensitization, asthma, and food allergy is consistent with previous *DOCK8* cohorts [10,11,12,13]. In the Turkish 20-patient series, atopic dermatitis was present in 18 of 20 patients and food allergy in 14 of 20; in the Mexican cohort, all patients had eczema and more than half had food allergy [11,12]. DOCK8-deficient CD4+ T cells show skewing toward a TH2 phenotype at the expense of TH1 and TH17 differentiation, while regulatory T-cell and B-cell tolerance defects may further amplify allergic inflammation and IgE production [20,21,22]. Severe atopy should therefore be viewed as an integral component of *DOCK8*-associated immune dysregulation rather than as an unrelated allergic comorbidity.

The NIH-HIES score in our cohort had been recorded during the initial evaluation for suspected HIES, before molecular confirmation of DOCK8 deficiency. Although previously reported in *DOCK8* cohorts, this score includes several features more characteristic of *STAT3*-HIES and is not validated as a *DOCK8*-specific measure of disease severity [10,24]. We therefore interpreted it descriptively. Its lack of significant association with survival, SCORAD, eosinophilia, or total IgE in our cohort further illustrates its limited *DOCK8*-specific interpretability, although these exploratory findings should be viewed cautiously given the small sample size.

Cutaneous involvement deserves particular emphasis because it was both universal and one of the earliest clinical clues in our cohort. This is consistent with previous *DOCK8* cohorts and the broader HIES spectrum, in which eczema and recurrent skin infections are major diagnostic clues. In the extended 64-patient *DOCK8* cohort, bacterial, viral, and fungal infections were frequent, skin abscesses and allergies were common, and HSV-related manifestations included skin infection, eczema herpeticum, and herpetic keratitis [10]. These observations support the view that skin disease in DOCK8 deficiency is not merely an accompanying dermatologic comorbidity, but rather a visible expression of impaired tissue immune surveillance, defective antiviral defense, barrier dysfunction, Th2 polarization, and Treg-related immune dysregulation [15,20,21,22,23,24,25,26,27]. Therefore, selected refractory cutaneous manifestations may require targeted antiviral or immunomodulatory approaches, including interferon-α for recalcitrant HPV disease and, in selected cases, type 2 cytokine blockade with dupilumab for severe dermatitis [28,29,30,31].

In our cohort, infectious complications remained a major source of morbidity, particularly recurrent respiratory infections, mucocutaneous candidiasis, and persistent cutaneous viral disease, consistent with established *DOCK8* phenotypes [4,9,10,14,15]. In particular, cutaneous viral manifestations, including herpetic keratitis, chronic cutaneous HSV infection, and HPV-related skin or mucosal disease, may be related to impaired antiviral surveillance resulting from actin cytoskeleton dysfunction, defective lymphocyte migration, and impaired cytotoxic synapse function [5,6,7,8]. In addition to these infectious manifestations, chronic pulmonary damage was prominent, with bronchiectasis representing the most frequent structural abnormality. Similar pulmonary complications have been reported in other *DOCK8* cohorts, highlighting the cumulative consequences of recurrent infection and delayed definitive treatment [11,12]. Pulmonary tuberculosis in one patient broadened the infectious spectrum but should be regarded as an uncommon rather than characteristic manifestation of DOCK8 deficiency. It has nevertheless been reported in selected cohorts and may reflect the combined effects of impaired cellular immunity, regional exposure, and chronic lung vulnerability [12].

Autoimmune and hematological manifestations occurred in a minority of patients but further illustrated the immune-dysregulatory spectrum of DOCK8 deficiency. Because the retrospective records did not consistently establish an immune-mediated mechanism for every cytopenia, we deliberately distinguished documented autoimmunity from nonspecific hematological abnormalities. Previous cohorts have described autoimmune hemolytic anemia, vasculitis, cytopenias, and other autoimmune or inflammatory complications in a subset of patients [9,10,13,14]. Experimental data showing impaired regulatory T-cell fitness, altered IL-2 signaling, defective immune synapse formation, and breakdown of peripheral B-cell tolerance provide a plausible mechanistic link between *DOCK8* actinopathy and autoimmunity [20,21,22].

Several less common manifestations were identified in our cohort. One patient had chronic liver disease with sclerosing cholangitis, a manifestation that has previously been reported in DOCK8 deficiency, sometimes in association with *Cryptosporidium* infection [25,26]. The giant aortic aneurysm observed in our cohort represents a rare but previously documented vascular manifestation of DOCK8 deficiency. Other vascular complications, including vasculitis, arterial stenosis, stroke, and aneurysmal disease, have also been described [10,32,33]. Chronic pancreatitis was another rare systemic manifestation in our cohort and is consistent with a previous report of recurrent pancreatitis in DOCK8 deficiency [34]. Collectively, these findings emphasize the broader systemic burden accompanying the classical infectious and atopic phenotype.

Malignancy occurred in two patients, underscoring cancer as an important complication of DOCK8 deficiency. Lymphomas and squamous cell carcinomas predominate among reported malignancies, whereas rarer neoplasms, including EBV-associated smooth-muscle tumors/leiomyosarcoma and microcystic adnexal carcinoma, have also been described [9,35]. One patient in our cohort developed cutaneous squamous cell carcinoma, a well-recognized *DOCK8*-associated malignancy. Notably, the other patient developed a GIST during childhood. Pediatric GIST is exceedingly rare and clinicobiologically distinct from adult GIST [36]. Its occurrence in a child with DOCK8 deficiency is therefore noteworthy and may broaden the recognized malignancy spectrum, although a causal association cannot be established from a single observation. Additionally, the occurrence of cancer in a pediatric series reinforces the need for long-term dermatologic, oncologic, and viral surveillance.

Large exon-level deletions were the predominant molecular finding in our cohort. This pattern aligns with the genomic architecture of the *DOCK8* locus at 9p24.3, where repetitive sequence elements predispose to large deletions and recombination-mediated rearrangements [4,9,10]. Importantly, exon-level copy-number variants may be missed by routine sequence-variant analysis of clinical or whole-exome sequencing data unless dedicated CNV analysis is performed. Therefore, in patients with a strong clinical suspicion of DOCK8 deficiency but negative or inconclusive sequencing results, targeted copy-number analysis using read-depth–based algorithms or complementary methods such as Multiplex Ligation-dependent Probe Amplification (MLPA) or array-based comparative genomic hybridization (CGH) should be considered [4,37].

HSCT remains the definitive treatment for DOCK8 deficiency. Most transplanted patients in our cohort had favorable outcomes, consistent with published pediatric and multicenter experience. The Turkish 20-patient series reported clinical and immunological improvement after HSCT and 91% survival among transplanted patients [11]. A multicenter HSCT study showed that transplantation can provide durable correction of the *DOCK8* phenotype across donor types, and the recent prospective trial reported survival without new *DOCK8*-related complications in most transplanted patients [16,17]. Mortality was markedly higher among patients who did not undergo transplantation; however, this crude comparison should be interpreted cautiously because of differences in transplantation timing and potential survivorship bias. In our cohort, non-transplantation was primarily related to donor unavailability or family decisions rather than inability to reach transplant evaluation due to rapidly progressive disease. Taken together with the established literature, these findings reinforce the importance of early diagnosis and timely transplant evaluation before severe irreversible complications develop.

This study has limitations. The retrospective design limited the completeness of selected clinical, genetic, and transplant-related variables. Although all patients had a molecularly confirmed diagnosis of DOCK8 deficiency, complete HGVS-level variant annotations were unavailable for two patients. In addition, HSCT procedures were performed at external transplant centers. Although clinically donor chimerism was sufficiently documented to support the classification of post-HSCT outcomes, detailed quantitative and longitudinal chimerism data were not consistently available. Likewise, detailed information on donor type and conditioning regimens was not available for all transplanted patients in our retrospective records. The sample size was small, as expected for a rare IEI, and subgroup comparisons, including analyses according to eventual HSCT status, should be interpreted as exploratory, with consideration of potential survivorship bias and differences in transplant eligibility and timing. Despite these limitations, our findings provide real-world pediatric data from a tertiary IEI center and reinforce the broad multisystem spectrum of DOCK8 deficiency.

## 5. Conclusions

Our pediatric cohort confirms the established infectious, atopic, and immune-dysregulatory phenotype of DOCK8 deficiency while highlighting uncommon hepatobiliary, vascular, and pancreatic manifestations. Gastrointestinal stromal tumor may represent a previously unreported association, although causality cannot be established. The favorable transplantation outcomes support existing evidence for timely molecular diagnosis and early HSCT evaluation.

## Figures and Tables

**Table 1:** Clinical phenotype of 17 patients with DOCK8 deficiency.

Patient ID	Sex	Parental Consang	Onset/Diagnosis Time (Mo)	NIH	SCORAD	Infections	Immune Dysregulation-Related Manifestations				Other Manifestations
							Allergic/Atopic Manifestations	Autoimmunity/Cytopenia	Lymphoproliferation/Organomegaly	Malignancy	
F1.P1	M	Yes	1/180	44	78	Recurrent pneumonia; skin infections; CMV	Eczema	AIHA; thrombocytopenia	Hepatosplenomegaly	No	Aortic aneurysm; growth retardation
F2.P1	F	Yes	5/37	23	54	Skin infections; candidiasis	Eczema; food allergy	—	Hepatomegaly	No	Growth retardation
F2.P2	M	Yes	4/6	19	54	Skin infections	Eczema; food allergy	—	—	No	Growth retardation
F3.P1	F	Yes	2/48	22	52	Recurrent pneumonia; skin infections; candidiasis	Eczema; specific IgE positivity	—	—	No	—
F4.P1	M	No	3/10	35	63	Recurrent pneumonia; skin infections	Eczema; asthma; food allergy; anaphylaxis	—	—	No	Growth retardation
F5.P1	M	No	6/78	36	35	Recurrent pneumonia; skin infections; candidiasis	Eczema; asthma	—	—	No	—
F6.P1	M	Yes	10/56	28	66	Recurrent pneumonia; skin infections (HPV papilloma); candidiasis	Eczema; asthma	—	—	Cutaneous SCC	Growth retardation
F7.P1	M	No	7/44	31	53	Recurrent pneumonia; skin infections	Eczema; asthma; food allergy	—	—	—	—
F8.P1	M	Yes	6/90	36	55	Recurrent pneumonia; skin infections; candidiasis	Eczema	—	Hepatomegaly	—	—
F8.P2	M	Yes	12/24	52	48	Recurrent pneumonia; skin infections; candidiasis; pulmonary tuberculosis	Eczema; asthma	—	Hepatomegaly	GIST	—
F9.P1	F	Yes	11/46	48	63	Recurrent pneumonia; skin infections; candidiasis; HPV-related perianal/perineal condyloma	Eczema	—	—	No	—
F10.P1	M	Yes	4/22	38	0	Recurrent pneumonia; skin infections; mucocutaneous candidiasis; herpetic keratitis	Eczema; asthma; food allergy; anaphylaxis	Neutropenia	—	No	—
F11.P1	M	Yes	10/40	42	55	Recurrent pneumonia; skin infections; candidiasis; CMV disease; herpetic keratitis; widespread herpes labialis	Eczema; asthma	—	—	No	Hypertension; chronic pancreatitis
F12.P1	F	Yes	12/50	30	50	Recurrent pneumonia; skin infections; CMV disease; chronic suppurative otitis	Eczema; asthma	Autoimmune hepatitis	Hepatosplenomegaly	No	Chronic liver disease/sclerosing cholangitis
F13.P1	M	Yes	1/12	18	52	Skin infections	Eczema	No confirmed	—	No	—
F13.P2	F	Yes	4/61	21	58	Skin infections; candidiasis; chronic HSV skin infection	Eczema	No confirmed	—	No	—
F14.P1	M	No	2/23	33	52	Recurrent pneumonia; CMV	Eczema; food allergy	No confirmed	—	No	—

Patient IDs represent 17 patients from 14 unrelated kindreds. P1 and P2 indicate affected individuals from the same kindred when applicable. Parental consang., parental consanguinity; Mo, months; NIH, National Institutes of Health clinical score; SCORAD, Scoring Atopic Dermatitis. — indicates absent. AIHA, autoimmune hemolytic anemia; CMV, cytomegalovirus; GIST, gastrointestinal stromal tumor; HPV, human papillomavirus; HSV, herpes simplex virus; SCC, squamous cell carcinoma.

**Table 2:** Hematological and immunological findings.

Patient ID	WBC	ANC	ALC	Peak AEC	PLT	MPV	IgG	IgA	IgM	IgE	CD3+	CD4+	CD8+	CD19+	NK
F1.P1	10.92	6.34	1.18	2.82	540	8.2	2990	108	48.6	>2000	340	213	111	515	301
F2.P1	19.08	3.73	3.94	14.16	447	8.5	1870	6.6	149	184	1250	352	589	980	130
F2.P2	14.62	5.96	5.25	33.72	542	8.7	694	25.5	26	>2000	1458	879	732	3174	256
F3.P1	8.90	3.30	2.00	4.20	317	8.3	1280	186	12.9	>2000	1318	646	654	572	88
F4.P1	15.68	5.16	5.30	26.00	330	9.6	535	88.8	30.7	>2000	1622	1336	159	3185	382
F5.P1	48.80	9.80	2.50	35.10	219	7.6	1290	118	121	1533	870	310	500	260	510
F6.P1	7.80	4.40	2.70	4.23	578	7.0	2120	625	37	116	1515	310	880	340	630
F7.P1	20.00	10.10	4.40	11.00	487	7.0	1200	104	28.1	3103	1984	977	836	1984	550
F8.P1	10.66	7.76	1.18	2.38	506	8.3	1330	586	23.3	>2000	858	303	434	219	127
F8.P2	13.02	8.73	2.64	2.14	352	7.2	1310	430	26.2	1156	1002	435	1235	204	1415
F9.P1	20.17	11.91	2.78	29.50	676	8.8	666	125	34	1471	1035	548	646	1186	623
F10.P1	11.90	1.00	5.06	4.33	332	7.9	1510	6.5	13.1	>2000	2868	1042	1578	1862	126
F11.P1	7.80	4.60	1.45	6.10	335	8.4	1380	429	27	3371	720	277	513	378	255
F12.P1	31.79	10.06	6.64	13.90	732	9.0	2450	177	42.7	1258	1818	649	1135	534	904
F13.P1	10.50	4.83	3.36	1.19	414	9.5	720	56.8	27.1	342	1465	1149	—	—	—
F13.P2	13.50	5.42	1.97	7.70	355	8.1	1330	148	38	790	902	542	292	642	353
F14.P1	11.73	2.70	3.56	6.46	407	10.6	401	46	48	947	3684	648	1984	823	279

WBC, ANC, ALC, AEC, and PLT are shown as ×10^3^/µL. MPV is shown as fL. IgG, IgA, and IgM are shown as mg/dL; IgE is shown as IU/mL. Values reported as >2000 IU/mL were analyzed as 2000 IU/mL for descriptive statistics. Lymphocyte subsets are shown as cells/µL. Peak AEC represents the highest eosinophil count recorded during follow-up; AEC values at initial evaluation are summarized separately in the text.

**Table 3:** Molecular genetic findings of 17 patients with DOCK8 deficiency.

Patient ID	Gene	Recorded Molecular Finding	Variant Category
F1.P1	*DOCK8*	Large deletion involving exons 2–7; c.(53+1_54-1)_(827+1_828-1)del	Large deletion/CNV
F2.P1	*DOCK8*	Large deletion involving exons 1–26	Large deletion/CNV
F2.P2	*DOCK8*	Large deletion involving exons 1–26	Large deletion/CNV
F3.P1	*DOCK8*	Large deletion involving exons 1–27	Large deletion/CNV
F4.P1	*DOCK8*	Homozygous c.(53+1_54-1)_(3234+1_3235-1)del, reported as a deletion involving exons 2–26	Large deletion/CNV
F5.P1	*DOCK8*	Large deletion involving exons 1–27	Large deletion/CNV
F6.P1	*DOCK8*	Homozygous C>G substitution at position 2715 of *DOCK8* cDNA, near the end of exon 22, resulting in p.Ser868Ter	Nonsense/stop-gain
F7.P1	*DOCK8*	Large deletion involving exons 1–27	Large deletion/CNV
F8.P1	*DOCK8*	Molecular diagnosis of DOCK8 deficiency documented; detailed variant annotation unavailable in the archived record	Not specified
F8.P2	*DOCK8*	Molecular diagnosis of DOCK8 deficiency documented; detailed variant annotation unavailable in the archived record	Not specified
F9.P1	*DOCK8*	C>G transversion at bp 2715 of *DOCK8* cDNA (NM_203447.3), near the end of exon 22, resulting in p.Ser868Ter	Nonsense/stop-gain
F10.P1	*DOCK8*	Homozygous c.4990T>C (p.Cys1664Arg)	Missense
F11.P1	*DOCK8*	Large deletion involving exons 1–22	Large deletion/CNV
F12.P1	*DOCK8*	A>T substitution in exon 46 at position 6068 of DOCK8 isoform 1 cDNA (NM_203447.3), resulting in p.Asp1986Tyr	Missense
F13.P1	*DOCK8*	Large deletion involving exons 1–26	Large deletion/CNV
F13.P2	*DOCK8*	Large deletion involving exons 1–26	Large deletion/CNV
F14.P1	*DOCK8*	Homozygous ENST00000453981.1:c.2206-2A>T	Splice-site variant

Variant descriptions are reported according to the available molecular genetic records. Molecular findings not originally provided in HGVS format are presented descriptively as recorded and were not retrospectively converted to HGVS nomenclature. CNV, copy number variant; HGVS, Human Genome Variation Society.

**Table 4:** Treatment, HSCT status, and prognosis.

Patient ID	Supportive/Medical Treatment	HSCT Status	Age at HSCT, Months	Final Status
F1.P1	Antibacterial/antifungal prophylaxis; IVIG	Not performed— no suitable donor	—	Deceased
F2.P1	Antibacterial/antifungal prophylaxis; IVIG	Performed (donor: MUD)	50	Alive; complete clinical recovery
F2.P2	Antibacterial/antifungal prophylaxis; IVIG	Performed (donor: MUD)	21	Alive; complete clinical recovery
F3.P1	Antibacterial/antifungal prophylaxis; IVIG	Performed (donor: NR)	68	Alive; complete clinical recovery
F4.P1	Antibacterial/antifungal prophylaxis; IVIG	Performed (donor: MRD)	29	Alive; complete clinical recovery
F5.P1	Antibacterial/antifungal prophylaxis; IVIG	Performed (donor: NR)	100	Alive; complete clinical recovery
F6.P1	Antibacterial/antifungal prophylaxis; IVIG	Not performed— no suitable donor	—	Deceased
F7.P1	Antibacterial/antifungal prophylaxis; IVIG	Performed (donor: MUD)	67	Alive; complete clinical recovery
F8.P1	Antibacterial/antifungal prophylaxis; IVIG	Performed (donor: MUD)	150	Deceased; acute GVHD
F8.P2	Antibacterial/antifungal prophylaxis; IVIG; antituberculosis treatment	Not performed— family declined HSCT	—	Alive
F9.P1	Antibacterial/antifungal prophylaxis; IVIG; IFN α-2b for HPV-related condyloma	Performed (donor: MUD)	90	Alive; complete clinical recovery
F10.P1	Antibacterial/antifungal prophylaxis; IVIG; acyclovir prophylaxis/treatment for herpetic keratitis	Performed (donor: NR)	44	Alive; complete clinical recovery
F11.P1	Antibacterial/antifungal prophylaxis; IVIG; acyclovir; IFN α-2b for widespread herpes labialis	Not performed— family declined HSCT	—	Deceased
F12.P1	Antibacterial/antifungal prophylaxis; IVIG	Not performed— no suitable donor	—	Deceased
F13.P1	Antibacterial/antifungal prophylaxis; IVIG	Performed (donor: NR)	18	Alive; complete clinical recovery
F13.P2	Antibacterial/antifungal prophylaxis; IVIG; acyclovir prophylaxis/treatment for HSV skin infection	Performed (donor: NR)	75	Alive; complete clinical recovery
F14.P1	Antibacterial/antifungal prophylaxis; IVIG	Performed (donor: MUD)	30	Alive; complete clinical recovery

HSCT, hematopoietic stem cell transplantation; GVHD, graft-versus-host disease; HSV, herpes simplex virus; HPV, human papillomavirus; IFN-α, interferon alfa; IVIG, intravenous immunoglobulin; MRD, matched related donor; MUD, matched unrelated donor; NR, not retrievable from the available records; —, not applicable.

**Table 5:** Summary of treatment and outcome data.

Variable	Result
Total patients	17
HSCT performed	12/17, 70.6%
Median age at HSCT	58.5 months, IQR 29.75–78.75, range 18–150
Complete clinical recovery after HSCT	11/12, 91.7%
Post-HSCT death	1/12, 8.3%
No HSCT	5/17, 29.4%
Mortality among non-transplanted patients	4/5, 80.0%
Relative risk (RR) of death, non-HSCT vs. HSCT	9.60, 95% CI 1.40–65.94
Overall survival	12/17, 70.6%
Overall mortality	5/17, 29.4%
Causes of death	Sepsis in 3 patients; ARDS in 1 patient; Acute GVHD in 1 patient

GVHD, Graft-versus-host disease, HSCT, hematopoietic stem cell transplantation; IQR, interquartile range; RR, relative risk; CI, confidence interval; ARDS, acute respiratory distress syndrome. Mortality analysis according to HSCT status was exploratory because of the small cohort size.

## Data Availability

The data that support the findings of this study are available on request from the corresponding author. The data are not publicly available due to privacy or ethical restrictions.
